# Genetic influence alters the brain synchronism in perception and timing

**DOI:** 10.1186/s12929-018-0463-z

**Published:** 2018-08-07

**Authors:** Victor Marinho, Thomaz Oliveira, Juliete Bandeira, Giovanny R. Pinto, Anderson Gomes, Valéria Lima, Francisco Magalhães, Kaline Rocha, Carla Ayres, Valécia Carvalho, Bruna Velasques, Pedro Ribeiro, Marco Orsini, Victor Hugo Bastos, Daya Gupta, Silmar Teixeira

**Affiliations:** 1 Neuro-innovation Technology & Brain Mapping Laboratory, Federal University of Piauí, Av. São Sebastião n° 2819 – Nossa Sra. de Fátima –, Parnaíba, PI CEP 64202-020 Brazil; 2 Genetics and Molecular Biology Laboratory, Federal University of Piauí, Parnaíba, Brazil; 3 The Northeast Biotechnology Network (RENORBIO), Federal University of Piauí, Teresina, Brazil; 4 Brain Mapping and Sensory Motor Integration Laboratory, Federal University of Rio de Janeiro, Rio de Janeiro, Brazil; 5 Master’s Program in Local Development and Health Sciences Applied Program, University Center Augusto Motta - UNISUAM, Rio de Janeiro, Brazil; 6 Brain Mapping and Functionality Laboratory, Federal University of Piauí, Parnaíba, Brazil; 7 Department of Biology, Camden County College, Blackwood, NJ USA

**Keywords:** Time perception, Genetic polymorphisms, Serotonin, Dopamine, Circadian rhythm, GABA

## Abstract

**Background:**

Studies at the molecular level aim to integrate genetic and neurobiological data to provide an increasingly detailed understanding of phenotypes related to the ability in time perception.

**Main Text:**

This study suggests that the polymorphisms genetic *SLC6A4* 5-HTTLPR, *5HTR2A* T102C, *DRD2/ANKK1*-Taq1A, *SLC6A3* 3’-UTR VNTR, *COMT* Val158Met, *CLOCK* genes and *GABRB2* A/C as modification factor at neurochemical levels associated with several neurofunctional aspects, modifying the circadian rhythm and built-in cognitive functions in the timing. We conducted a literature review with 102 studies that met inclusion criteria to synthesize findings on genetic polymorphisms and their influence on the timing.

**Conclusion:**

The findings suggest an association of genetic polymorphisms on behavioral aspects related in timing. However, order to confirm the paradigm of association in the timing as a function of the molecular level, still need to be addressed future research.

## Background

The interindividual differences in the time perception are evident in daily actions and their neurobiological aspects (i.e. walking, talking, reward tasks, executive functions, and cognition), with neurotransmitters acting in synchronization of the Central Nervous System (CNS). Neurotransmitters actions help to synchronize stimuli [1, 2], and trigger responses on time scales that range from the milliseconds (motor coordination), seconds to minutes (conscious time perception) and hours of the day (circadian rhythms) [3, 4]. It has been known that the genetic makeup enables the coding of different environmental stimuli, modulated through the action of the serotonergic, dopaminergic, GABAergic neurotransmission as well as circadian oscillations [5].

The hypothesis that the genetic polymorphisms in the expression of neurotransmitter systems and in circadian rhythm influences the perceptual capacity [6], promoted an increase in the number of genetic research to understand the molecular underpinnings of the temporal processing. To evaluate the genetic factors in temporal processing, Balci et al. [7] and Meck et al. [8] analyzed the effects of *5HTR2A*, *SLC6A4*, *SLC6A3* and *DRD2* expression on peak interval (PI) or non-reward procedures in rats. Results showed that rats genetically knockout for *5HTR2A*, *SLC6A4*, *SLC6A3*, and *DRD2* have less precision in the performance of PI activities when compared to control rats. This supports the importance of the role of several gene products in the phenotypic behavior associated with processing time intervals.

Genotypic investigations regarding brain function associated with timing are passive to determination of endophenotypes [9]. The literature seeks to trace a genetic profile of association with psychometric functions and behavioral performances, for example, fear conditioning, timing tasks by means of visual, tactile or auditory stimuli in humans or other species (Table 1) [10–12]. In principle, human cognitive performance is highly variable and under strong genetic control. Moreover, qualitative or quantitative genetic changes promote underestimation or overestimation of time according to the Scalar Expectancy Theory (SET) [5, 13, 14]. This is accounted by the interference in the number of oscillations captured per time unit from the internal clock [15, 16], and judgments of time intervals may result from changes in the pulse flow from an internal pacemaker in the presence of an event [17]. Consequently, genetic polymorphisms increase or decrease the speed of the internal clock, modulating the neurotransmission of the pulses and reactions to stimuli that determine the synchronism in motor and cognitive activities [11, 13, 18].

**Table 1:** Summary of genetic studies investigating the impact of time perception

Genotyping	Protocol	Stimulus	Duration	Results
*SLC6A3* 5-HTTLPR	Group: 273 young healthy. Cognitive tasks: (memory and Face Identity Perception tasks).	Visual Face images	Seconds range, varied for each participant.	There was neither an association between the 5HTTLPR genotype and cognitive tasks, but there might be a tendency for better performance of SL as compared with SS carriers for fEP [24].
*SLC6A4* 5-HTTLPR, *5HT2A* T102C, *DRD2/ANKK1*-Taq1A, *SLC6A3* 3’-UTR VNTR, *COMT* Val158Met, *MAOA* VNTR, and *CLOCK* genes.	Group: 647 healthy individuals, Questionnaire. Cognitive test. Production and Discrimation tasks.	Visual Auditory	1 s, 3 s, 6 s, 12 s, 15 s	Stability of an individual’s temporal accuracy and precision across in supra-second intervals (ranging from 3 s to 15 s) in the cognitive tasks and time perception tasks. Female participants are more likely to underestimate time production task when an explicit counting strategy is not employed [6].
*COMT* Val158Met, *5HTR2A* T102C.	Group: 90 healthy Japanese. Cognitive tasks and fMRI study.	Visual	2 s, 3 s	Results demonstrate that the *COMT* genotypes are related to recognition accuracy, whereas the *5HTR2A* genotypes are associated with RTs for recognition. In addition, strong connectivity in the cingulo-frontal networks is closely linked to a better working memory performance, regardless of the genotypes [98].
*COMT* Val158Met, *SLC6A3* 3′UTR VNTR and *DRD4* exon 3 VNTR.	Group: 52 healthy Estonians. Cognitive tasks and Discrimination task.	Visual	23 ms, 70 ms, 105 ms, 735 ms,	*SLC6A3* variability no showed difference in study. *COMT* Val158Met and *DRD4* exon 3 VNTR differ in their effects on attentional functions as explicated in long-SOA metacontrast [20].
*DRD2/ANKK1*-Taq1A.	Group: 25 healthy individuals. Temporal or color Discrimination task and fMRI acquisition	Visual	350 ms, 400 ms, 450 ms, 550 ms, 600 ms, 650 ms; 1,4 s, 1,8 s, 2 s, 2,2 s, 2,4 s, 2,6 s.	A1 carriers of the Taq1A polymorphism exhibited worse performance on temporal task. However, greater activation in the striatum and right dorsolateral prefrontal cortex, as well as reduced volume in the cerebellar [22].
*DRD2/ANKK1*-Taq1A and *COMT* Val158Met.	Group: 41 healthy individuals. Time perception tasks. Fixed-intervals tasks.	Visual	10s, 17 s.	*DRD2/ANKK1*-Taq1A in the striatum and *COMT* Val158Met, affecting the breakdown of dopamine in the prefrontal cortex— to interval timing and reward magnitude modulation of decision thresholds [7].
*COMT* Val158Met, *SLC6A3* 3’-UTR VNTR.	Group: 95 healthy individuals. 64-channel EEG study. Continuous performance test.	Visual	450 ms, 600 ms, 750 ms, 900 ms.	Effects of *SLC6A3* and *COMT* on the occipito-temporal activity in CNV. In addition, there was a trend towards an interaction between the two polymorphisms [19].
*SLC6A3* +/+ rats *SLC6A3* −/− rats *SLC6A3* +/− rats	Group: DAT-mutant rat. Peak interval task. Administration: Methamphetamine hydrochloride.	Visual	15 s, 20s, 45 s, 140 s, 200 s.	Complete loss of temporal control and altered sensitivity to drugs. Lower threshold for initiating responding in the timing task [8].
*DRD2/ANKK1*-Taq1A and *COMT* Val158Met.	Group: 65 healthy individuals. Temporal discrimination task and motor tempo task.	Visual	500 ms, 2 s	*DRD2/ANKK1*-Taq1A (A1+ allele) was associated with variability for the 500 ms duration only, whereas the *COMT* Val158Met (Val/Val) was associated with variability for the 2000 ms duration only. Additionally, the *DRD2/ANKK1*-Taq1A was associated with slower preferred motor time [21].
*SLC6A3* +/+ rats *SLC6A3* −/− rats *SLC6A3* +/− rats	Group: KD rat and WT rat. Peak interval task and administration of the Raclopride.	Visual	20s, 40s, 60s, 80s, 100 s, 120 s.	DAT KD rats responded at higher levels in peak trials than WT rats in all conditions, but particularly during the fixed-interval 30 peak trials [1].
*SLC6A4* 5-HTTLPR, *5HT2A* T102C, *DRD2/ANKK1*-Taq1A, *SLC6A3* 3’-UTR VNTR and *COMT* Val158Met.	Group: 44 healthy individuals. Discrimination task.	Visual Auditory	Combinations of supra seconds.	No differences between time representation and dopamine-genes. However, show association between serotinine-related genes and parameters derived from psychometric functions PSE [13].
*DRD2/ANKK1*-Taq1A	Group: Transgenic rat C57BL/6-CB. Peak interval tasks.	No	Fixed-interval: 24 s.	Overexpression of D2 receptors in the striatum caused a reduction in operant response rate, a broadening of the distribution of operant responses in time and an increase in the latency of the peak in response rate, consistent with an impairment in timing accuracy. The progressive ratio operant task confirmed that D2 overexpressing rats exhibited reduced operant motivation [23].

*fEP* face identity perception, *RT* response time, *SOA* Stimulus onset asynchrony, *CNV* Contingent negative variation, *KD* Knockdown, *WT* Wild type, *PSE* Point of subjective equality

Therefore, the individual variability in the coding of the time intervals resulting from single nucleotide polymorphisms (SNPs) and numbers of tandem repeat variations (VNTRs) modifies timing process [19, 20]. In summary, studies suggest that the *SLC6A4* 5-HTTLPR, *5HTR2A* T102C, *DRD2/ANKK1*-Taq1A, *SLC6A3* 3’-UTR VNTR, *COMT* Val158Met, *CLOCK* genes and *GABRB2* A/C (rs6556547) expression in the cortical and subcortical areas influence the time judgment. Thus, it becomes relevant for neurogenetics to elucidate endophenotypes associated with the perception of the sub-second and supra-second intervals [6, 11, 13, 18, 21].

Finally, we presented a state-of-the-art for genetic polymorphisms and time perception, highlighting the main discoveries, and addressing further directions for clinically relevant neuroscience research. Although we have some papers not directly related to genetic influence on the time perception; these additional studies demonstrate behavioral phenotypes related to cognition and executive function, both have a key role in time intervals synchronization. These studies support neurochemical changes caused by differential gene expression, as well as a relationship with cognitive modulations embedded in ability to synchronize the time intervals.

### Genetic of the serotoninergic system on the neurobiological aspects inbuilt in timing

Analyses of neural and genetic mechanisms have contributed to the understanding of how the time-trial process occurs [19]. Sysoeval, Tonevitsky and Wackermann [13] investigated differences in the neurobiological basis in perceiving stimuli through polymorphisms analysis (*SLC6A4* 5-HTTLPR, *5HTR2A* T102C, *DRD2/ANKK1*-Taq1A, *SLC6A3* 3’-UTR VNTR and *COMT* Val158Met) concomitantly with time discrimination tasks. Since the results do not reveal consistencies in data from the studies of genetic polymorphisms related to the dopaminergic system, it may be inferred that increased or decreased serotonin expression also influences the processing of time intervals. The results demonstrated that chemical modulations in the CNS in perceptive activities relates to serotonin concentration in the coding second to minutes duration intervals. Genetic polymorphisms in neurotransmission predispose participants to different behavioral phenotypes, and consequently we infer inappropriate recruitment, decrease or increase of neurotransmitters levels, which causes modulations of neural inputs in the timing circuits.

In particular, a biomarker of serotoninergic expression related to individual variability in cognition, the *SLC6A4* gene located in 17q11.2, encode a transmembrane protein that transports serotonin from synaptic spaces. An In/Del polymorphism exists within the 5′ region flanking the regulatory region of the *SLC6A4* gene, a highly polymorphic region (5-HTTLPR) associated with levels of transport and serotonin uptake. The genotypes related to *SLC6A4* 5-HTTLPR, the short allele (S) show decreased serotonin reabsorption compared to the long allele (L) [25, 26]. The allelic differences are associated with a decrease in the availability of serotonin. Individuals carrying the short allele have increased extracellular serotonergic levels compared to those with two copies of the long allele [27]. Fallgatter et al. [10] confirmed that individuals with one or two copies of the *SLC6A4* 5-HTTLPR short allele have greater brain activity and less error in memory and timing stimulus activities. Therefore, Heinz et al. [28] reported that the healthy carriers of short alleles of the *SLC6A4* 5-HTTLPR have increased amygdaloid neuron activation and the connection of neural inputs between the amygdala and the ventromedial prefrontal cortex than homozygous individuals for the long allele. It has been shown that genetic variations that encode serotonin play a central role in social learning neurobiology, the system of emotions and decision making.

Additionally, neurochemistry influences working memory, which is a system of limited capacity that permeates almost all levels of cognition, ranging from perceptive awareness to intelligence [29]. Thus, cortical and subcortical signaling is influenced by the receptors differential expression of receptors in regions of the brain that are involved in perceptual processing and executive functions. Accordingly, Crisan et al. [30] investigated the effects of *SLC6A4* 5-HTTLPR polymorphism concomitant with neuroimaging tools, through tasks that measure decision-making on economic risks in healthy financial market volunteers. The results of genotyping in the 5-HTTLPR region in this retrospective study indicate that carriers of the short alleles, same in heterozygosity, exhibit better performance in financial activities and a lower percentage of economic losses compared to homozygotes for the long allele. The relationship of *SLC6A4* 5-HTTLPR polymorphism with variable serotonin expression has been associated with neuropsychiatric conditions [11], social anxiety disorder [31], aggressiveness [32], depression [33] and impulsivity [34], diseases that contribute to the deficit in the time perception. However, genetic studies associated with cognitive parameters are not only linked to *SLC6A4* 5-HTTLPR [35], but the studies by Burt and Mikolajewski [36] show that that subjects make less harmful decisions, besides an adjuvant action of variations in the *5HTR2A* gene on cognitive aspects.

The *5HTR2A* gene located at 13q14–21 encodes the expression of postsynaptic type 2A (5-HT2A) serotonin receptors, which signal via diacylglycerol and triphosphate inositol during serotonergic stimulation. The *5HTR2A* gene influences the perceptual capacity of stimuli and executive functions [36], and are associated with impulsivity disorders in motor and cognitive planning tasks, likely from deficient connections as well as a decrease of serotonergic receptors in brain regions that perform these activities (i.e. prefrontal cortex and limbic system) [36, 37]. The *5HTR2A* T102C polymorphism corresponds to a thymine (T) shift by a cytosine (C) at position 102 of the codon in the promoter region of the gene and determines changes in the amino acid coding that make up the 2A receptors. Genotypes with TT alleles have higher expression of 5-HT2A than homozygous CC and heterozygous TC [38]. The genotypes studies at a molecular level in conjunction with pharmacological models suggests that the *5HTR2A* T102C polymorphism modulates the timing activity of visual stimuli in work memory tasks [39]. The ability to influence the perception of visual stimuli is because 5-HT2A receptors are highly expressed in the visual cortex [40]. Thus, the alterations in the density of the occipital cortex due to genetic variations decrease the expression of 5-HT2A, which facilitate the appearance of deficiencies in time interpretation and in risk of neurological diseases [41, 42].

### Genetic aspects of the dopaminergic system in timing

The control in the human ability to judge the time intervals originated of the external stimuli has as one of the bases, the dopaminergic system [11, 43]. Among the genes that influence dopaminergic neurotransmission, the *DRD2* gene located in 11q23.2, encodes a G protein-coupled receptor in postsynaptic neurons, playing a key role in neurotransmission, besides influencing several behavioral phenotypes [44, 45]. In particular, SNP *DRD2/ANKK1*-Taq1A triggers glutamate changes by lysine (Glu713Lys) in *ANKK1*, altering the function of the *DRD2* promoter region and expression of the D2-type receptors. In view of the foregoing, two alleles (A1 and A2) can be identified, the presence of one or two alleles A1 is associated with the reduction of 20 to 40% of the D2 receptor in areas of the striatum, region essential in the encoding of neural inputs in the timing [13, 46]. The transient expression of D2 receptors have been demonstrated to cause a deficit in neural mechanisms in the indirect pathway activation of the prefrontal cortex, motor cortex, and premotor, consequently, thus impairs the acquisition of temporal control, and results in underestimation during timed-motor tasks [7].

Furthermore, molecular influence on dopaminergic neurotransmission in presynaptic terminals is mediated by *SLC6A3* gene located in 5p15.3, important to the chemical regulation between dopaminergic circuits and pathways related to cognition and time perception [47]. In this context, the polymorphism of the repeats in tandem *SLC6A3* 3’-UTR VNTR located in the 15 exon of the 3′ untranslated region, contains serial repetitions of 40 bp ranging from 3-repeats (3R) to 13-repeats. (13R), and the 9-repeats (9R) and 10-repeats (10R) series are the most common alleles in the general population. Consistent with the dopamine transporter (DAT) functionality, carriers of a 9R allele exhibit lower DAT protein encoding in the prefrontal cortex when compared to their 10R homozygous homologues. Although the protein structure is not altered, it is thought that function is affected by regulating mRNA stability, transport and protein synthesis [47]. The functional alleles 10R and 9R alter the related phenotypes with planning and execution processes of working memory tasks, stimuli perception and increase the risk of neurological diseases [48–50] because of patients with a 9R allele exhibit less DAT activity than their homozygotes 10R. In the prefrontal cortex and caudate nucleus, and thus modulate dopamine levels in the CNS during the processing of temporal and cognitive information [51, 52].

The studies are also based on the influence of dopamine-degrading enzymes, especially the *COMT* gene located in 22q11, which encodes dopaminergic degradation in the synaptic cleft. The *COMT* gene encodes the enzyme catechol-o-methyltransferase (COMT enzyme) that catalyzes the transfer of a methyl group from S-adenosylmethionine to catecholamines [11]. The level of degradation is modified by means of the *COMT* Val158Met polymorphism, known to influence enzymatic activity in the prefrontal cortex, increasing the dopaminergic levels and thus deregulating the nigrostriatal circuit in the time delay of supra seconds. Carriers of the Met allele (L) are more visualized in time-stamping studies by means of motor and cognitive tasks in patients with Parkinson’s disease [18]. Carriers of the Met allele (L) have relatively low COMT enzyme activity and, presumably, have greater availability of dopamine. While the carriers of the Val allele (H) have a relatively high activity of COMT enzyme, and less availability of dopamine [11, 53]. The *COMT* Val158Met polymorphism has been associated with impairments in working memory [11], emotional problems [54], reduced attention levels [20], and associated with a risk of neurological diseases [55, 56]. Thus, the influence on the perception of time due to these neurological disorders is presumed to act in brain areas responsible for coding processes of time intervals, and in cognitive functions embedded in the timing.

Bartholomew et al. [6] have shown the hypothesis of genetic polymorphisms influencing the time interpretation and express complex human phenotypes. The authors reported that genetic polymorphisms contribute to individual variability in neurobiological aspects at internal clock speed. In the study methodology, 647 healthy individuals were analyzed in production and discrimination of time intervals, however, only 148 individuals were examined based on Genome-wide association study (GWAS) to see if any genetic polymorphism is associated with a trait. GWAS typically focus on associations between SNPs and human traits for different phenotypes in timing. The authors used the *DRD2/ANKK1*-Taq1A, *SLC6A3* 3’-UTR VNTR, and *COMT* Val158Met, which are correlated with precision in judging of time intervals. The genotypes were chosen based on the minor allele frequency (MAF) greater than 5%, and thus, the neurobiological data related to MAF identified common variations that may contribute to the understanding of the human behavioral phenotype in time coding.

Meck et al. [8], based on molecular influence on dopaminergic neurotransmission, the authors assessed behavioral phenotype in knockout rats for the *SLC6A3* and *COMT* gene and under the influence of drug. The authors concluded that the circuits of the prefrontal cortex and striatum have less activation during the encoding of stimuli, which is due to changes in the neurotransmitter levels due alterations in COMT enzyme and DAT. On the basis of this, we propose that the genetic polymorphisms *SLC6A3* 3’-UTR VNTR and *COMT* Val158Met alter the internal clock speed by decreasing accuracy in judging time intervals. Thus, the neural substrates underlying the three clock stages (clock phase, memory phase, and decision) can be modulated according to the genetic polymorphisms, which is likely due to changes in dopaminergic pathways. According to this model, dopamine participates mainly in the clock phase. An increase in dopaminergic signaling accelerates the accumulation of impulses over time and modifies the coding process of accumulated information, and consequently distorts judgment time [5].

Transport, receptors and dopaminergic degradation have been shown to be critical for the time interval in humans and animals [57]. Experiments with genetically knockout mice for *SLC6A3* and *DRD2* have increased levels of dopamine in the prefrontal cortex and neural inputs deficient in neurotransmitter via the nigrostriatal pathway. Elevated levels of the neurotransmitter imply impairment both in the time interval and in the motivation to work for food rewards [23]. In association with these characteristics, it was shown that deficits in time accuracy appear to be mediated by deficiencies in the systems of motivation, working memory or sustained attention. In general, the overall alteration of DAT and D2 receptor expression through *SLC6A3* 3’-UTR VNTR and *DRD2/ANKK1*-TAq1A results in underestimation in timing of behavior. It is inferred that the resulting effect does not resemble the effect of clock speed induced by injections of drug dopaminergic agonists when rats are trained by peak interval (PI) [58].

### Influence of clock genes in the timing

The innate preference in the morning or evening is one of the characteristics determined by the circadian rhythm phase. From this circadian synchronization, our brain as an efficient machine in the orchestration of time scales encodes physiological information and cognitive behaviors [58, 59]. In mammals, this system is organized by a central clock located in the suprachiasmatic nucleus (SCN) of the hypothalamus, in addition to a series of peripheral oscillators present in the liver, lungs, adrenal glands, and other tissues [59–61].

The SCN regulates the biological rhythms of the organism through self-regulated transcription-translation loops of the so-called *CLOCK* genes in a 24-h period, with the *CLOCK*, *ARNTL*, *PER* (1, 2 and 3) and *CRY* (1 and 2) [62, 63]. Briefly, the CLOCK/ARNTL protein complex expresses negative regulators PER1–3 and CRY1–2 proteins, since they inhibit the expression of the CLOCK/ARNTL heterodimer. The CLOCK/ARNTL heterodimer controls the genetic expression acting in the physiology of the organism and, thus, the modulation of heterodimer levels leads to the rhythmicity of various metabolic and cognitive functions [59, 64, 65].

The control of CLOCK-ARNTL heterodimer levels exhibits time-dependent fluctuations, and thus modulate the synchronization capability of shorter intervals, such as second-to-minutes [65, 66]. Agostino and Cheng [58] provided experimental evidence showing changes in dopaminergic levels in rat striatum as a function of the circadian cycle, with lower levels of dopamine during the day and a peak at night. This occurs due to regulators in the promoter region of the *SLC6A3*, *DRD1* and *MAOA* genes, demonstrating that the expression of these components is linked to cycles of luminosity and metabolic variations [58]. Rats with modulations in the dopaminergic, levels in the dorsal striatum for lack of melatonin have an impact on the stimuli perception in fixed interval tasks. In addition, the lack of melatonin has been associated with circadian clock components, focusing on the *PER2* gene. The genetic expression of *PER2* is involved in the circadian regulation of dopaminergic metabolism through dorsal striatum and substantia nigra pars compacta (SNpc) oscillations [58].

Therefore, it is increasingly accepted that the variations in genetic components of circadian rhythm affect the rhythmicity of the organism in timing as well as environmental changes [59, 67]. In exemplification, workers who abruptly change their work shift may present mood problems, social and work activities, as well as favor the development of deficient cognitive functions [68, 69]. It has been shown that the *PER2* gene effect on insomnia was relatively stronger than environmental factors such as high stress at work. Thus, it is suggested that the genetic susceptibility of the individual should be considered for the sleep problems control [70]. In the context, Song et al. [71], in a Korean population, demonstrated that in addition to the involvement of the *PER2* gene, a synergistic action with other circadian genes may increase the risk of the diurnal preference toward evening [71]. Given the influence of genetic variations related to the circadian rhythm on sleep homeostasis, it is easier to understand how endogenous rhythmicity influences the interval timing, as changes in the sleep-wake cycle influence the processing of shorter time intervals. Späti et al. [66], analyzed the time perception of individuals who experienced sleep deprivation, and their findings showed that sustained wakefulness distorts the timing interval through pacemaker pulse oscillations over a 24-h period, with an exponential increase and saturation in the rate of the pacemaker with time constant of 18.2 h.

Neural synchronism deficient in the coding of the time intervals led by circadian dysregulation was studied by means of time estimation tasks with rats under conditions of 24 h in the dark (D/D) or 24 h in the daylight (L/L), different of the normal homeostatic condition of 12 h light / 12 h dark (L/D) [72]. Agostino et al. [72] observed that the group trained in the DD condition showed higher precision, whereas in the L/L condition the rats lost temporal control. This evidence is important in view of the fact that the light is the main cue for circadian rhythm. The time perception, in addition to varying with the time of day, is strongly correlated with circadian variations in body temperature and serum melatonin levels [73]. The circadian rhythm is indicated as a regulator of the interval timing by means of modulations in the dopamine levels, which is implicated as a key neurotransmitter responsible for the pacemaker-accumulator operation [67]. In fact, the processing of timing interval involves the interaction of cortico-striatal circuits through the dopaminergic-glutamatergic pathways. With this, alteration in dopaminergic neurotransmission during the day caused by circadian dysfunctions strongly influences the timing interval. However, the changes in neurotransmitter levels were shown to be reversed by levodopa injections in rats with circadian timing disruptions. So, it is suggested that a daily increase in dopamine is required for the accurate performance of timekeeping tasks [74].

Polymorphisms in different genomic regions are not only responsible for the stable changes throughout the organism, but also have the potential to modulate neural coding for interval timing [75]. In summary, the circadian genes expression in the striatum and substantia nigra, important structures involved in temporal processing and dopaminergic neurotransmission, influence neural oscillations when L/L conditions are induced [74]. If on the one hand, circadian changes alter the expression of dopamine and consequently the timing interval, dopamine-associated polymorphisms have also been implicated in circadian changes, showing a clear synergism between two systems [76].

### Genetic of the GABAergic system have an impact on neurobiological aspects inbuilt in the time perception

**Fig. 1:**
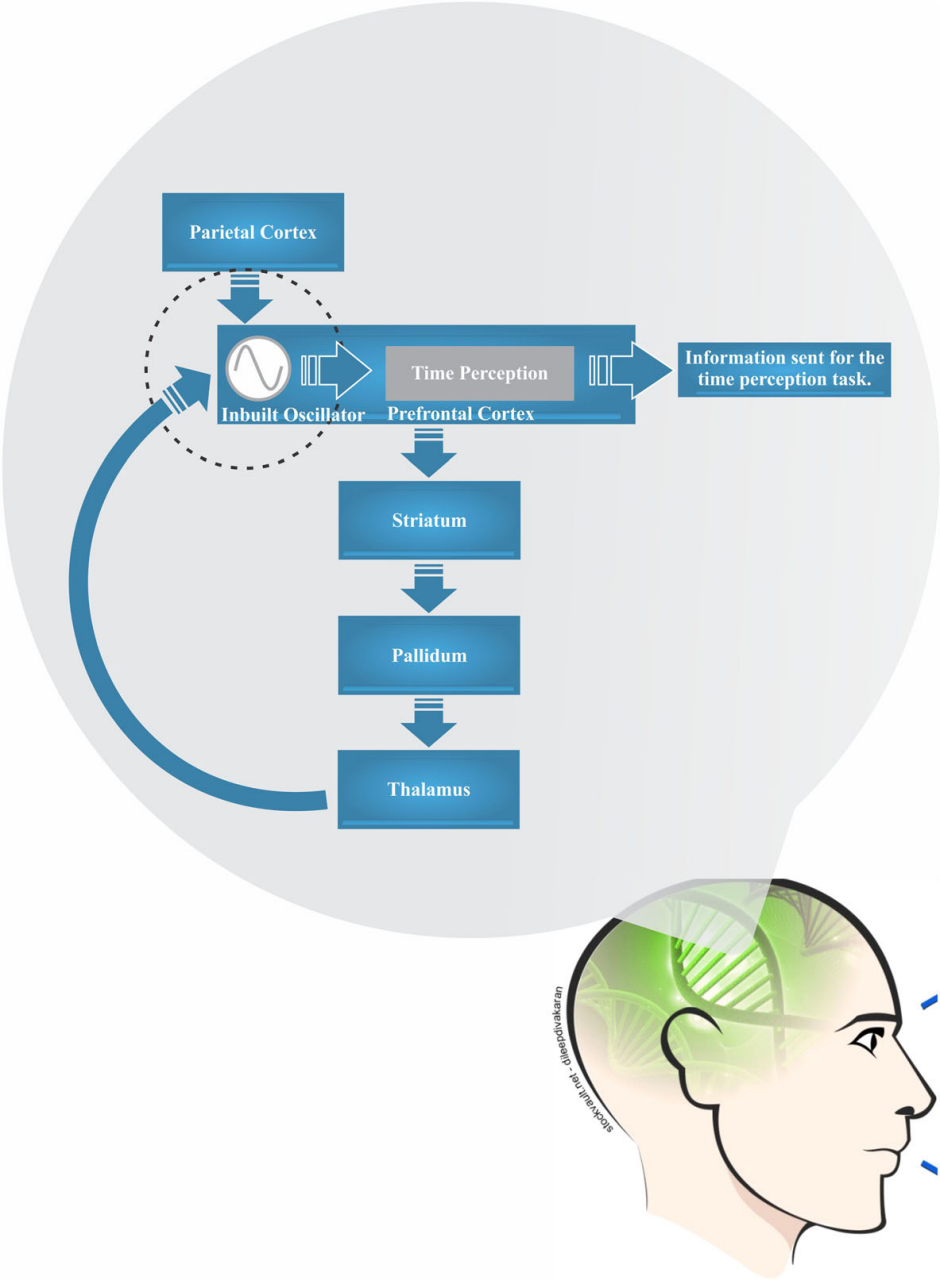
Modular connection among the parietal cortex, prefrontal cortex and striatum. The proposed inbuilt modular clock in the prefrontal cortex is responsible for synchronizing the time perception. Genetic polymorphisms modulate the functions of the parietal and prefrontal cortex; reduced interference in the role of striatal oscillations in the representation of time intervals in the brain. Thus, decreases the efficiency in coding the time intervals

The β2 GABA_A_ subunit receptor is a multiple subunit chloride channel receptor that mediates the action of gamma-aminobutyric acid (GABA), a fast inhibitor of synaptic transmission in the CNS. The receptor is expressed by the *GABRB2* gene, located at 9q22.1-q22.3, which plays a role in modulating synapses and maintains the excitation-inhibition balance in the brain [77]. Different genetic polymorphisms alter the expression or function of elements of GABAergic neurotransmissions, such as the SNP *GABRB2* A/C type intronic polymorphism that decrease the expression of messenger RNA (mRNA) required for the synthesis of β2 subunit. This polymorphism decreases GABA concentration at the postsynaptic level and has thus been associated with changes in memory and attention, both influencing time-interval processing [5, 78]. It has been argued that *GABRB2* A/C modulates β2 subunit activity of the GABA_A_ receptor in various time interval regions, specifically the visual cortex, frontal cortex, dopaminergic circuit associations and striatal-thalamic-cortical [79]. In this sense, it modifies the temporal processing of information obtained from the activity of sensory receptors, which is responsible for capturing and redirecting information to be interpreted in executive actions proposed in the classical pacemaker-accumulator model, this is consistent with the role of neural oscillations in synchronizing parts of distributed modular clocks in the brain (Fig. 1) [80–84].

Experimental evidence suggests that GABAergic fast inhibitory neurotransmission – mediated by GABA_A_ receptor subtype – plays a key role in beta oscillations as shown by a study of the primary motor cortex of rats [85]. Furthermore, linkage and association mapping between genetic polymorphisms (microsatellite and single-nucleotide polymorphisms) shows significant dependence of beta-range oscillation phenotypes with *GABAA* gene location in chromosome 4 [85, 86]. More experimental evidence directly implicates GABAergic transmission in timing functions of the brain. In a psychophysics study, measuring visual interval estimation and reproduction, it was shown that the elevated levels of GABA in the visual cortex lead to the underestimation of visual intervals [77].

Kononowicz and Van Rijn [87] showed that subjective timing of millisecond intervals does not depend on changes indexed by the evoked potential known as contingent negative variation, and that the subjective experience of time is better reflected by distinct features of evoked potentials occurring after offset of comparison interval in a time estimation task [87]. In addition, the authors demonstrated that the neural responses evoked from GABAergic connections predicted the subjective perception of the comparison interval, with evoked responses more similar to the normal interval, independently of the duration presented [87, 88]. Furthermore, experimental evidences by means of computational modeling demonstrate GABAergic neurons inhibitory to the production of gamma oscillations in electroencephalography [89], as well as the GABA performance in the visual cortex for the differences in the individual phenotypes, as consequence of the decrease of the threshold of synchronization of visual stimuli [77, 87, 88].

Van Loon et al. [89] analyzed differences in the processing of visual stimuli due to the synchronism of GABAergic neurons, implying, therefore, that GABA acts as a mediator in the perception of visual intervals. Thus, experiments with pharmacological modeling of GABAergic agonists and antagonists in accordance with genomic analyses are suggested to elucidate the role of GABAergic system in time discrimination tasks [77, 90]. Furthermore, GABAergic activity is susceptible to the modulation of time perception and the polymorphic form *GABRB2* A/C and its haplotypes have the capacity to influence the risk of neurological diseases (i.e., Parkinson’s Disease, Schizophrenia, Bipolar Disorder), which affect the processing of time intervals by the brain in laboratory-controlled tasks or in the performance of everyday activities [78, 91]. In addition, the study by Oblack, Gibbs, and Blat [92] has shown that the neurobiological aspects related to the perceptual capacity of time in autistic subjects is deficient, which is associated with elevations of GABAergic activity in the visual cortex and auditory cortex. Moreover, these results may reveal underestimation of the time in relation to the timing of the stimuli [89, 90].

Another aspect of the GABA as an inhibitor of synaptic transmission in the CNS, is demonstrated through postsynaptic polarization, and decrease of intracellular Ca_2+_ levels in the medium spiny neurons. This occurs by means of GABAergic loops that are also important for interval timing mechanisms, among which are the glutamic acid decarboxylase (GAD), an enzyme that converts glutamate to GABA. The *GAD1* gene encodes the GAD67 in both the cell body and nerve terminals. GAD65, on the other hand, is encoded by the *GAD2* gene and is restricted to the nerve terminals [78, 93]. It can be hypothesized that reduced inhibitory transmission in the brain may play a role in timing based on the level these two isoforms, GAD65 and GAD67. Reduced synthesis of GABA due to inhibition or decreased levels of GAD may result in neural inputs deficits inbuilt in neurobiological aspects in timing, such as stimulus perception, decision-making and memory. Clearly, a dysregulation of glutamate and GABA metabolism in the brain may contribute to neurological diseases that affect the time judgment (for example, Anxiety disorders and Depression) [77, 93].

Few studies have evaluated the hypothesis of the genetic interaction of *GABRB2* A/C on the interindividual variability of behavioral phenotypes related to a timing error in healthy individuals or in patients with some neurological disease. However, few state-of-the-art demonstrates studies studied electrophysiological measures of neural firing in monkeys the GABAergic performance in the coding and timing of stimuli [94–96]. Interestingly, they showed that the perceived duration is a consequence of the magnitude of the neuronal response at visual time-intervals in GABAergic circuits. The findings suggest a link between electrophysiological results due to inhibition of the specific sensory excitatory activity of the optical pathways of visual stimulation [89, 97, 98]. It may be that the prediction of the error as a function of the perceptual capacity is determined by local calcium limitations in the activation of the GABA receptors in the visual cortex, which affects the excitation-inhibition equilibrium [99].

### Limitations of existing research

Some factors, such as sensory modality, intensity, size, complexity, familiarity are not standardized in the various studies of the time perception, making it impossible to completely determine the association with genetic polymorphisms in the neural inputs during time coding. Limitations involved on population size and further studies with larger samples are needed to determine whether such genetic polymorphisms alone may or may not help explain the interindividual differences in timing. A limitation of this study, are few works that relate genetics on time perception, in particular, the variation referring to the neurotransmitter GABA. The presented studies suggest an applicability of the *GABRB2* variants in the timing since it modifies in the inbuilt oscillator in executive functions essential in the judgment of the time.

## Conclusion

The study of genetic polymorphisms is not only relevant to health, but also to various behavioral phenotypes that alter the temporal judgment. Thus, more studies are needed to substantiate the genotypic contributions on performance in temporal activities, by understanding the neurobiological mechanisms at the molecular and biochemical level behind timing synchronism. In summary, levels of neurotransmitters such as serotonin and dopamine are regulated by *SLC6A4*, *5HTR2A*, *DRD2/ANKK1*, *SLC6A3*, and *COMT* gene that modulate the chemistry of the neural bases in the prefrontal circuits [100], associated with studies of cognition and working memory [101], learning and repetitive motor tasks in relation to a visual stimulus [102]. Regarding the *CLOCK* genes, corticosteroid changes due to alterations in *PER2* expression promoted impacts in the time interval, as it acts on the dopaminergic metabolism due to the influence of circadian variations induced by changes in the light / dark cycle.

Furthermore, few studies have analyzed the *GABRB2* A/C polymorphism in time perception tasks, and our hypothesis of acting on time intervals is supported by changes in the processing of perceptual process information in the visual cortex, suggested by changes in neural oscillations. In circadian pacemakers, for example, the SCN, due to limitations in the activation of GABA receptors in the visual cortex, affects the excitation-inhibition balance in the visual stimuli perception. Thus, genetic and cognitive information provide the means that direct the mechanisms underlying the perceptual behavior of time in humans.

## Abbreviations

5-HTTLPR: Highly polymorphic region
CNS: Central Nervous System
CNV: Contingent negative variation
COMT enzyme: Enzyme catechol-o-methyltransferase
DAT: The dopamine transporter
fEP: Face identity perception
GABA: Gamma-aminobutyric acid
GAD: Glutamic acid decarboxylase
GWAS: Genome-wide association study
KD: Knockdown
MAF: Minor allele frequency
mRNA: Messenger RNA
PI: Peak interval
PSE: Point of subjective equality
RT: Response time
SCN: Suprachiasmatic nucleus
SET: Scalar Expectancy Theory
SNpc: Substantia nigra pars compacta
SNPs: Single nucleotide polymorphisms
SOA: Stimulus onset asynchrony
VNTRs: Numbers of tandem repeat variations
WT: Wild type
